# TNF blockade induces a dysregulated type I interferon response without autoimmunity in paradoxical psoriasis

**DOI:** 10.1038/s41467-017-02466-4

**Published:** 2018-01-02

**Authors:** Curdin Conrad, Jeremy Di Domizio, Alessio Mylonas, Cyrine Belkhodja, Olivier Demaria, Alexander A. Navarini, Anne-Karine Lapointe, Lars E. French, Maxime Vernez, Michel Gilliet

**Affiliations:** 1 Department of Dermatology, University Hospital CHUV, Lausanne, 1011 Switzerland; 2 Department of Dermatology, University Hospital of Zurich, Zurich, 8091 Switzerland

## Abstract

Although anti-tumor necrosis factor (TNF) agents are highly effective in the treatment of psoriasis, 2–5% of treated patients develop psoriasis-like skin lesions called paradoxical psoriasis. The pathogenesis of this side effect and its distinction from classical psoriasis remain unknown. Here we show that skin lesions from patients with paradoxical psoriasis are characterized by a selective overexpression of type I interferons, dermal accumulation of plasmacytoid dendritic cells (pDC), and reduced T-cell numbers, when compared to classical psoriasis. Anti-TNF treatment prolongs type I interferon production by pDCs through inhibition of their maturation. The resulting type I interferon overexpression is responsible for the skin phenotype of paradoxical psoriasis, which, unlike classical psoriasis, is independent of T cells. These findings indicate that paradoxical psoriasis represents an ongoing overactive innate inflammatory process, driven by pDC-derived type I interferon that does not lead to T-cell autoimmunity.

## Abstract (web summary)

The pathogenesis of paradoxical psoriasis in patients receiving anti-TNF treatments for classical psoriasis is unclear. Here, the authors show that anti-TNF drugs enhance the production of type I interferon by plasmacytoid dendritic cells, causing skin lesions that, unlike classical psoriasis, lack T- cell autoimmunity.

## Introduction

Tumor necrosis factor (TNF) is a homotrimeric cytokine produced by immune and epithelial cells in response to infection or tissue injury^1,2^. TNF exerts potent pro-inflammatory functions via activation of immune cells and vascular endothelial cells^2–4^. Increased TNF expression levels can be found at sites of inflammation in many autoimmune diseases, such as rheumatoid arthritis, Crohn’s disease, or psoriasis^5–7^. TNF blockade is highly efficacious and has become the benchmark in management of these diseases^8–11^. As such, more than two million patients have been treated with TNF blockers.

Nevertheless, TNF blockade as a therapeutic option has its limitations. Long-term TNF neutralization increases susceptibility to infections and skin cancer^12,13^. Another common side effect of TNF blockade is the development of inflammatory skin lesions, which resemble psoriasis and are observed in 2–5% of patients receiving anti-TNF therapy^14–18^. These skin manifestations are called “paradoxical psoriasis”, as TNF blockade is usually highly efficacious in psoriasis treatment. Notably, this side effect even occurs in patients undergoing successful psoriasis treatment with anti-TNFs. More severe cases necessitate interruption or complete cessation of anti-TNF therapy and, for several diseases, no equivalent alternative treatments exist. Therefore, understanding the pathogenic mechanism underlying paradoxical psoriasis, and its distinctions from classical psoriasis, remains a critical issue for the future design of successful therapeutic and preventive measures.

Classical psoriasis is a chronic, autoimmune skin disease mediated by T cells^19–21^. Evidence for a pathogenic role of T cells stems from the following observations: first, T-cell-targeted therapies, including cyclosporine (inhibition of calcineurin in activated T cells), DAB-IL-2 (interleukin-2 receptor-specific fusion toxin)^22^, and inhibitors of T-cell costimulation, including alefacept^23^, efalizumab^24^, and CTLA-4-Ig^25^, are efficacious in psoriasis treatment; second, *HLA-Cw6* represents the strongest genetic risk variant associated with psoriasis^26^; third, clinically relevant xenotransplant models of psoriasis are dependent on T cells^27–29^; and, finally, lesional T cells are oligoclonal and recognize epidermal autoantigens^30–34^. These pathogenic T cells mediate the chronic and relapsing course of psoriasis and define it as an autoimmune disease.

Autoimmune T-cell responses in psoriasis are initiated by a subset of dendritic cells called plasmacytoid dendritic cells (pDCs), which infiltrate pre-psoriatic skin and are activated to produce type I interferons (IFNs)^35^. pDC-derived type I IFNs unleash the autoimmune response by promoting activation and maturation of conventional DCs (cDCs) that stimulate expansion of autoreactive T cells. These autoreactive T cells—in particular CD8^+^ T cells—migrate into the epidermis, where they recognize keratinocyte autoantigens and induce keratinocyte hyperproliferation^28,36^. Whether paradoxical psoriasis follows a similar pathomechanism remains unknown.

Here we show that paradoxical psoriasis induced by anti-TNF is characterized by an exaggerated type I IFN response, which does not lead to T-cell autoimmunity. Anti-TNF antibodies directly increase the capacity of pDCs to produce type I IFNs, by inhibiting their maturation. The exaggerated type I IFN response induced by anti-TNF treatments is sufficient to trigger a psoriatic skin phenotype. However, in contrast to classical psoriasis, type I IFN fails to induce cDC maturation and the subsequent activation of autoimmune T cells that is required for a chronic-relapsing disease course. Thus, paradoxical psoriasis is a side effect of an anti-TNF treatment stemming from an overactive, but self-limiting innate inflammation driven by pDC-derived type I IFN.

## Results

### Clinical characterization of paradoxical psoriasis

**Fig. 1:**
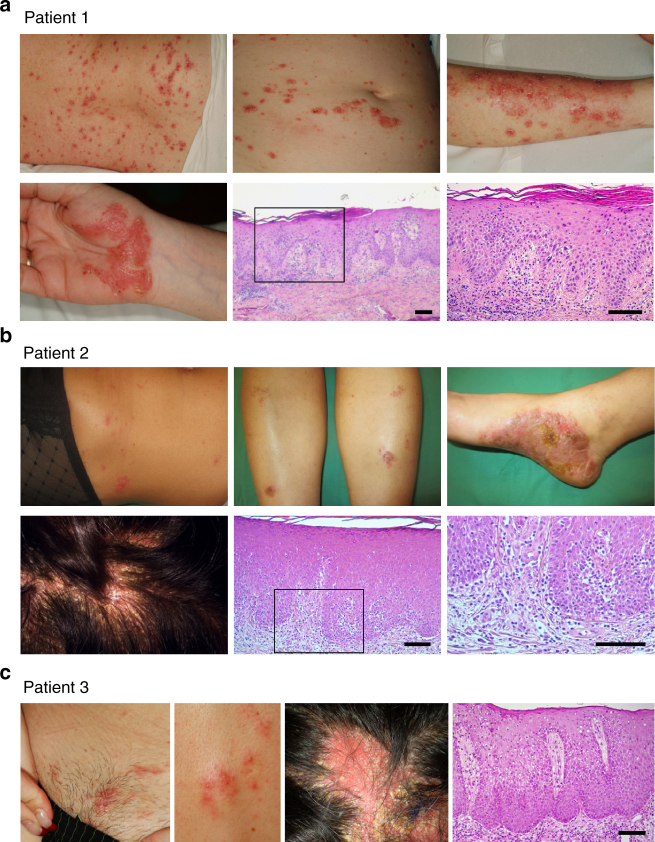
Clinical and histological presentation of paradoxical psoriasis. **a**–**c** Photographs of cutaneous lesions and corresponding histopathology of three individual patients presenting paradoxical psoriasis. **a** Patient 1 with small erythemato-squamous plaques disseminated over the entire body resembling guttate psoriasis and palmoplantar psoriasis-like lesions. Histology with a classical psoriasis pattern with acanthosis, papillomatosis, parakeratosis, and loss of the granular layer, but with spongiosis. **b** Patient 2 with partially crusted, eczematiform lesions on her legs and trunk, palmoplantar psoriasis-like lesions, and severe scalp involvement. Histology with acanthosis, papillomatosis, also in addition to spongiosis and minimal interface dermatitis. **c** Patient 3 with small erythematous plaques and pustules. Noncicatricial alopecia on the site of scalp involvement. Histology with acanthosis, papillomatosis, and spongiosis. Clinical signs and histopathology of the patients shown are representative of the patient population in this study

We analyzed 25 paradoxical psoriasis patients as summarized in Supplementary Table 1. Mean age of the patients was 44.8 years (range 15–73 years). Mean duration of anti-TNF treatment until onset of paradoxical psoriasis was 9.5 months (range 3 weeks to 5 years). Anti-TNF therapy indications include Crohn’s disease (*n* = 6), psoriasis and/or psoriatic arthritis (*n* = 8), ankylosing spondylitis (*n* = 8), rheumatoid arthritis (*n* = 1), as well as SAPHO (*n* = 1) and juvenile rheumatoid arthritis (*n* = 1). Patients were treated with the anti-TNF antibodies infliximab (*n* = 10), adalimumab (*n* = 10), certolizumab (*n* = 1), and golimumab (*n* = 2), and the TNF-receptor fusion protein etanercept (*n* = 2). Anti-TNF-induced paradoxical psoriasis appeared independent of the underlying diseases or the type of anti-TNF agent used (Supplementary Table 1). Paradoxical psoriasis regressed in all patients when anti-TNF therapy was discontinued, but relapsed or persisted in 7 of 11 cases (64%) when anti-TNF treatment resumed. These relapses occurred despite switching to another anti-TNF agent. Importantly, no relapses were seen upon discontinuation of anti-TNF treatment, which suggests that paradoxical psoriasis does not represent de novo psoriasis. The clinical presentation showed great variations reminiscent of classical psoriasis in its clinical forms (plaque-type, guttate, and pustular) or particular sites of involvement (palmoplantar, scalp, and skin folds; Fig. 1 and Supplementary Table 1). However, we also observed some clinical particularities of paradoxical psoriasis, including a higher frequency of palmoplantar involvement as compared to classical psoriasis (80 vs. 2–19%^37,38^) and severe noncicatricial alopecia, in numerous cases with scalp involvement (Fig. 1). Histopathology of paradoxical psoriasis showed a large spectrum with three identifiable patterns: an eczematiform spongiotic pattern; a psoriasis-like pattern (with different amounts of intraepidermal or subcorneal neutrophilic infiltration); and a lichenoid pattern with focal interface dermatitis (Fig. 1). However, these patterns were usually overlapping, presented at variable degrees in most cases, and did not correlate to the clinical presentations. These findings suggest that paradoxical psoriasis is a transient side effect induced by TNF blockade independent of treatment type (class effect) with diverse clinical and histological presentations resembling psoriasis.

### High IFN expression and pDC numbers in paradoxical psoriasis

**Fig. 2:**
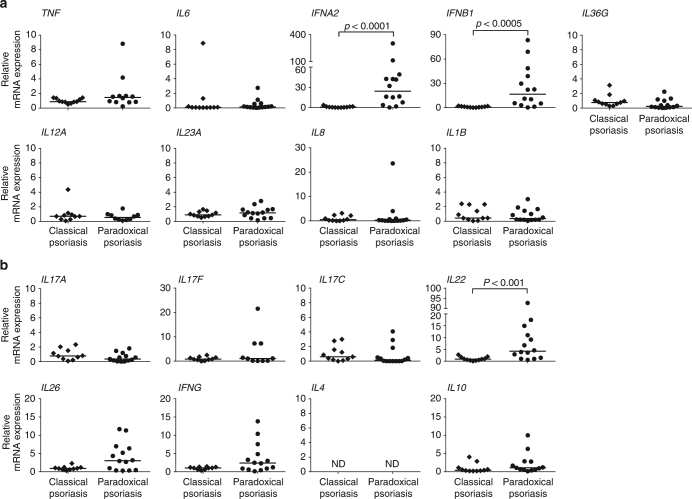
Increased type I interferon in skin lesions of paradoxical psoriasis. **a** mRNA expression analysis of pro-inflammatory cytokines *TNF*, *IL6*, *IFNA2*, *IFNB1*, *IL36G*, *IL12A*, *IL23A*, *CXCL8*, and *IL1B* relative to *GAPDH* in skin lesions of paradoxical psoriasis compared to classical plaque psoriasis. **b** mRNA expression analysis of adaptive T-cell-derived cytokines *IL17A*, *IL17F*, *IL17C*, *IL22*, *IL26*, *IFNG*, *IL4*, and *IL10* relative to *GAPDH* in skin lesions of paradoxical psoriasis as compared to classical plaque psoriasis. Dots represent individual patient and horizontal bar denotes the median value. Data shown as mRNA expression level relative to mean expression in classical psoriasis (mean value for classical psoriasis was set at 1). Statistical analysis was performed with unpaired non-parametric Mann–Whitney *U*-test. ND = not detected

We analyzed mRNA expression levels of selected innate cytokines involved in the pathogenesis of psoriasis to identify expression patterns unique to paradoxical psoriasis. We observed no significant difference in the expression levels of *TNF*, *IL23A*, *IL12A*, *IL36G*, *IL8* (*CXCL8*), *IL6*, and *IL1B* when comparing skin lesions from paradoxical psoriasis with classical psoriasis (Fig. 2a). In contrast, type I IFNs *IFNA2* and *IFNB1* expression was greatly increased in paradoxical psoriasis relative to chronic plaque psoriasis (Fig. 2a). Importantly, high levels of type I IFN expression were observed in all samples, despite the variability in clinical and histological presentation. Thus, uniform high levels of type I IFN expression in lesional skin characterize anti-TNF-induced paradoxical psoriasis. Interestingly, adaptive T-cell-derived cytokines *IL17A*, *IL17F*, *IL17C*, *IL26*, *IFNG*, *IL4*, and *IL10* show comparable levels in skin biopsies from paradoxical and classical psoriasis (Fig. 2b). However, we found significantly increased *IL22* expression in paradoxical psoriasis, which correlated significantly with the increased type I IFN expression (*IFNA2* *r* = 0.567, *p* < 0.005; *IFNB1* *r* = 0.474, *p* = 0.017; calculated by Spearman’s rank-correlation).

**Fig. 3:**
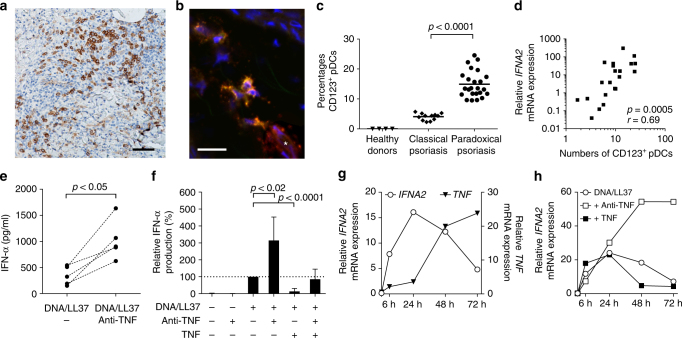
Plasmacytoid dendritic cell (pDC)-derived type I interferon controlled by TNF. **a** Representative immunohistochemical CD123 (IL3RA)-staining of skin from a patient with paradoxical psoriasis. **b** Representative confocal laser scanning microscopy of paradoxical psoriasis stained for BDCA2 (CLEC4C) (green), CD123 (red), and DAPI (blue) shows pDCs co-staining for BDCA2 and CD123 (yellow) and CD123 single-positive endothelial cells (*, red). **c** Histological quantification of CD123-positive pDCs per total dermal infiltrate in skin from healthy donors, psoriasis, and paradoxical psoriasis. **d** Correlation of numbers of CD123-positive pDCs with gene expression of *IFNA2*. **e** IFN-α produced by pDCs enriched from peripheral blood mononuclear cells of healthy volunteers 48 h after stimulation with DNA/LL37 complexes with or without addition of anti-TNF antibodies. **f** Relative amount of IFN-α produced by pDCs from healthy volunteers at 48 h, unstimulated or upon stimulation with DNA-LL37 complexes with or without anti-TNF antibodies, with or without addition of TNF. **g** Relative *IFNA2* and *TNF* mRNA expression by pDCs isolated from healthy volunteers, stimulated with DNA/LL37, and kept in culture for 6, 24, 48, or 72 h, respectively. **f** Relative *IFNA2* mRNA expression by pDCs from healthy volunteers 6, 24, 48, and 72 h upon stimulation with DNA/LL37 complexes either with anti-TNF antibodies or addition of TNF. Dots represent individual patient/healthy donor (**c**, **d**) and horizontal bar denotes the mean value (**c**). Data in **f** depicted as relative expression (percentage) over amount of IFN-α produced upon stimulation with LL37/DNA (set at 100%); data shown as mean ± SD of six independent experiments with blood from six healthy volunteers (for DNA/LL37 + anti-TNF + TNF; *n* = 3). Data in **g** and **h** depicts one representative of four independent experiments with cells from four different healthy individuals. Statistical analysis was performed in **c** with unpaired Student’s *t*-test and in **e** and **f** with paired Student’s *t*-test, in **d** the Spearman’s rank-correlation coefficient was calculated

Type I IFNs are preferentially expressed by pDCs, or natural type I IFN-producing cells. They can produce 50- to 100-fold more type I IFNs than any other cell type. We therefore investigated whether pDCs are present in paradoxical psoriasis skin lesions by staining paraffin-embedded sections with CD123 (IL3RA). CD123^+^ lymphoid cells in skin represent bona-fide pDCs^35^, as demonstrated by co-staining with BDCA2 (CLEC4C) in selected cryo-samples of paradoxical psoriasis (Fig. 3b). pDCs were absent in normal skin from healthy volunteers. However, confirming previous studies^39^, we found large numbers of pDCs in paradoxical psoriasis skin lesions (Fig. 3a). This increase was significantly greater than the number of pDCs found in classical plaque psoriasis (Fig. 3c). Expression of both *IFNA2* (Fig. 3d) and *IFNB1* (Supplementary Figure 1) significantly correlated with pDC quantity, suggesting that they represent the principal source of type I IFN. Notably, pDC accumulation coincides with elevated type I IFN expression at a uniform rate regardless of the clinical or histological phenotype in paradoxical psoriasis.

### Anti-TNF enhances IFN by inhibiting pDC maturation

Given the increased *IFNA2* expression in anti-TNF-induced paradoxical psoriasis, we investigated whether TNF blockade would enhance IFN-α production by pDCs directly. As LL37 complexed with DNA has been shown to activate pDCs in psoriasis^40,41^, and because *CAMP* mRNA expression (corresponding to LL37) in paradoxical psoriasis was comparable to psoriasis (Supplementary Figure 2), we used LL37/DNA complexes as stimulus to activate enriched human peripheral blood pDCs in the presence or absence of anti-TNF antibodies. TNF blockade significantly enhanced IFN-α production by stimulated pDCs measured 48 h after stimulation (Fig. 3e, f). This was direct effect of TNF blockade and not mediated by Fc-receptors as shown by a similar IFN-α increase when using certolizumab, a Fc-free Fab-fragment of a monoclonal antibody, but not an irrelevant human IgG antibody (Supplementary Figure 3a, b). Furthermore, addition of recombinant TNF to the culture strongly suppressed IFN-α production by activated pDCs (Fig. 3f) indicating that TNF controls IFN-α production by pDCs. To gain further insights into the mechanisms by which TNF controls IFN-α production, we performed time course analyses of cytokine expression in activated pDCs. *IFNA2* expression occurred early, peaking at 24 h, whereas *TNF* expression increased at later time points (48 and 72 h after stimulation) and coincided with the decrease of *IFNA2* expression (Fig. 3g). Anti-TNF antibodies did not affect early *IFNA2* expression but markedly increased its levels at 48 and 72 h, indicating that TNF blockade prolongs the ability of pDC to produce type I IFNs (Fig. 3h and Supplementary Figure 4). Moreover, addition of recombinant TNF to the culture shortened *IFNA2* expression by pDCs (Fig. 3h). Together these data show that IFN-α precedes TNF expression and suggest that TNF replaces IFN-α by inhibiting its expression. Because TNF drives pDC differentiation into mature DCs, which lose their ability to produce IFN-α^42^, we hypothesized that anti-TNF would prolong type I IFN production of activated pDC by inhibiting their maturation. Indeed, anti-TNF significantly decreased maturation of pDCs as shown by reduced surface expression of HLA-DR (CD74) 48 h after activation (Suppl Figure 5a, b). Anti-TNFs also reduced expression of co-stimulatory molecules CD80 and CD86, as well as maturation marker CD83, on activated pDCs (Supplementary Figure 5c–g). Addition of recombinant TNF, which suppressed IFN-α production by pDCs, strongly upregulated expression of CD80, CD86, and CD83 (Supplementary Figure 5c–g). These data suggest that TNF controls the duration of IFN-α production by promoting differentiation of pDCs into mature DCs. Consequently, TNF blockade inhibits pDC maturation and prolongs their ability to produce IFN-α, providing an explanation for high levels of type I IFN in anti-TNF-induced paradoxical psoriasis.

### Anti-TNF increases IFN and pDC numbers in the skin

**Fig. 4:**
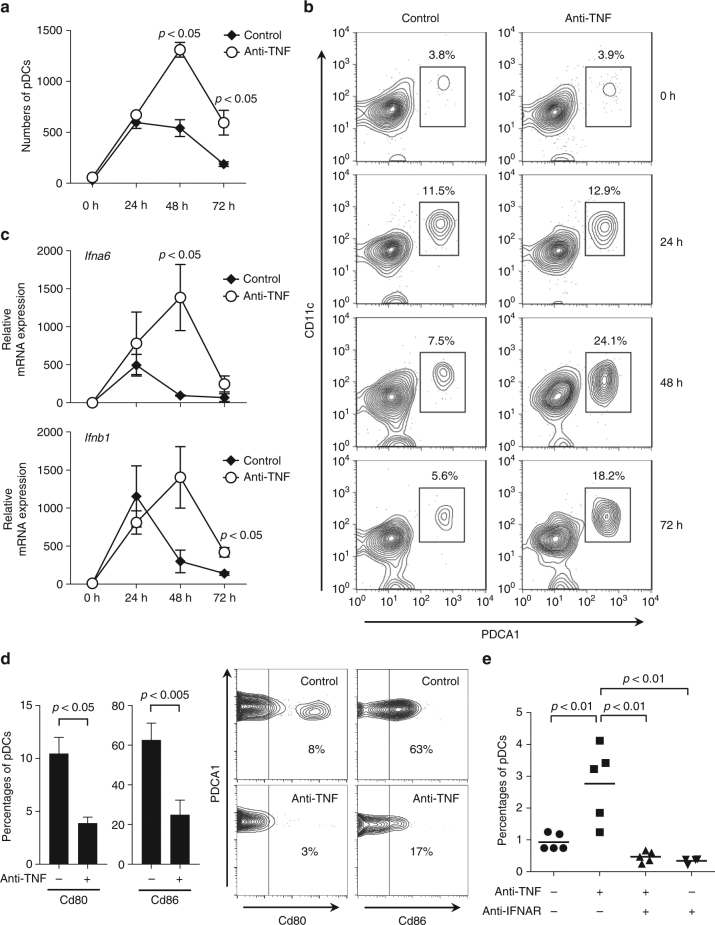
Effect of anti-TNF treatment on plasmacytoid dendritic cell (pDC)-activation in skin. **a** pDC numbers infiltrating the skin upon mechanical injury of the back of mice treated with or without anti-TNF. pDCs quantified by flow cytometry at indicated time points. **b** One representative contour plot for each group at indicated time points. **c** Total skin mRNA expression of the type I interferons *Ifna6* and *Ifnb1* upon mechanical injury of mice treated with or without anti-TNF at indicated time points. **d** Expression of co-stimulatory molecules Cd80 and Cd86 on skin-infiltrating pDCs 48 h after mechanical skin injury of mice treated with or without anti-TNF. **e** Percentage of pDCs infiltrating the skin of mice upon mechanical injury in the presence or absence of anti-TNF and/or anti-IFNAR antibodies. Experiment depicted in **a** and **c** is representative for at least three independent experiments using at least three mice per group. Bar charts in **d** show mean values plus SEM of six mice, with pDCs from skin of two mice pooled for each data point; one representative contour plot for each group (two mice pooled) is depicted in the right panel **d**. All statistical analyses were performed with unpaired Student’s *t*-test

Next, we studied whether anti-TNFs are sufficient to increase type I IFN production in vivo utilizing a skin injury mouse model. In this mouse model, repetitive tape stripping leads to a short-lived pDC infiltration into injured skin, peaking at 24 h and declining at 48 h (Fig. 4a)^43^. Anti-TNF treatment promoted significant increased and sustained pDC infiltration (Fig. 4a, b), which paralleled prolonged type I IFN expression (Fig. 4c). Importantly, pDC depletion largely abrogated this type I IFN expression, which confirmed in vivo that pDCs are the principal source of type I IFNs following TNF blockade (Supplementary Figure 6). Similar to the human in vitro data, TNF blockade in vivo significantly inhibited pDC maturation as shown by a decreased expression of Cd80 and Cd86 (Fig. 4d). Interestingly, blocking type I IFN signaling by an anti-type I IFN-receptor (anti-IFNAR) antibody significantly reduced the numbers of pDCs infiltrating injured skin (Fig. 4e). As CXCR3 ligands CXCL9, CXCL10, and CXCL11 are induced by type I IFNs and mediate pDC migration into the skin^44,45^, we analyzed their expression in our mouse model. Indeed, we found a significant, type I IFN-dependent overexpression of *Cxcl10* and *Cxcl11* in the skin of anti-TNF treated mice at 24, 48, and 72 h, as anti-IFNAR treatment completely abrogated their expression (Supplementary Figure 7). These data show that type I IFN production sustains skin infiltration of pDCs and suggest an amplification loop in which type I IFN produced by pDC promotes additional pDC infiltration into the skin. These data demonstrate that blocking TNF decreases pDC maturation and enhances type I IFN production by pDCs to amplify their skin infiltration.

### Anti-TNF promotes paradoxical psoriasis via IFN

**Fig. 5:**
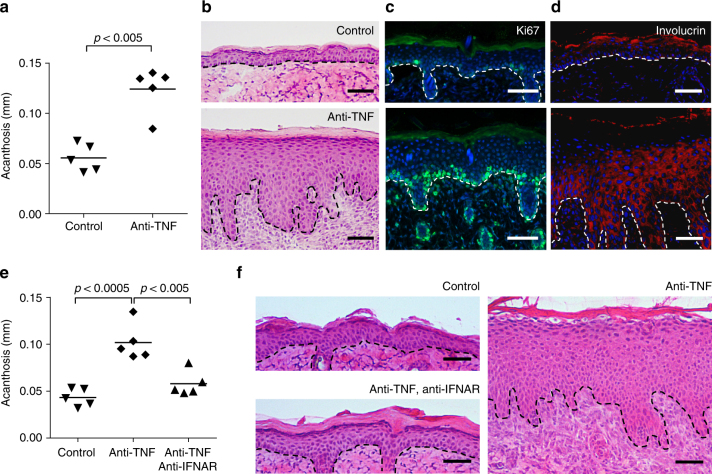
Type I interferon-dependent psoriasis-like skin phenotype induced by anti-TNF. **a** Quantification of epidermal thickening (acanthosis) in mice treated with or without anti-TNF antibody 7 days after mechanical injury by tape stripping. Representative HE staining (**b**), immunofluorescence staining for Ki-67 (proliferation, **c**), and involucrin (differentiation, **d**) for both untreated and anti-TNF-treated mice 7 days after mechanical injury. **e** Quantification of acanthosis 7 days after mechanical injury of mice treated with or without anti-TNF, and with or without anti-IFNAR antibodies. **f** Representative HE staining of skin 7 days after mechanical injury of untreated mice and mice treated with anti-TNF antibody alone or anti-TNF and anti-IFNAR antibodies combined. Experiment depicted is representative for at least three independent experiments. Scale bars represent 50 μm. Dashed line **b**–**d** and **f** represents border between epidermis above and dermis below. All statistical analyses were performed with unpaired Student’s *t*-test. Anti-IFNAR = anti-type I interferon receptor antibody

Transient type I IFN production by pDCs promotes wound healing^43^, whereas sustained expression may initiate classical psoriasis development^35,46^. Because anti-TNF treatment in wild-type mice increases pDC accumulation and type I IFN production in the skin, we determined whether it also induced a psoriasis-like phenotype. Indeed, 6–7 days after tape stripping, the epidermis of anti-TNF treated mice showed typical hallmarks of psoriasis, including acanthosis, parakeratosis, and a focal loss of the granular layer. In addition, we observed basal and suprabasal Ki67 expression indicative of keratinocyte hyperproliferation and involucrin expression throughout the entire epidermis suggesting abnormal keratinocyte differentiation (Fig. 5a–d). In contrast, the epidermis of control mice was similar to untreated skin showing minimal Ki67-positive keratinocytes and involucrin expression within the upper epidermal layers (Fig. 5a–d). We then treated mice with anti-IFNAR antibodies to determine if enhanced type I IFN induced the psoriatic phenotype. Inhibition of type I IFN signaling decreased the anti-TNF-induced psoriatic phenotype to levels indistinguishable from control mice (Fig. 5e, f). Together, these data indicate that anti-TNF induces a psoriatic phenotype through enhanced and sustained type I IFN production by pDCs. These data provide a mechanism that underlies paradoxical psoriasis.

### Development of paradoxical psoriasis is T-cell-independent

**Fig. 6:**
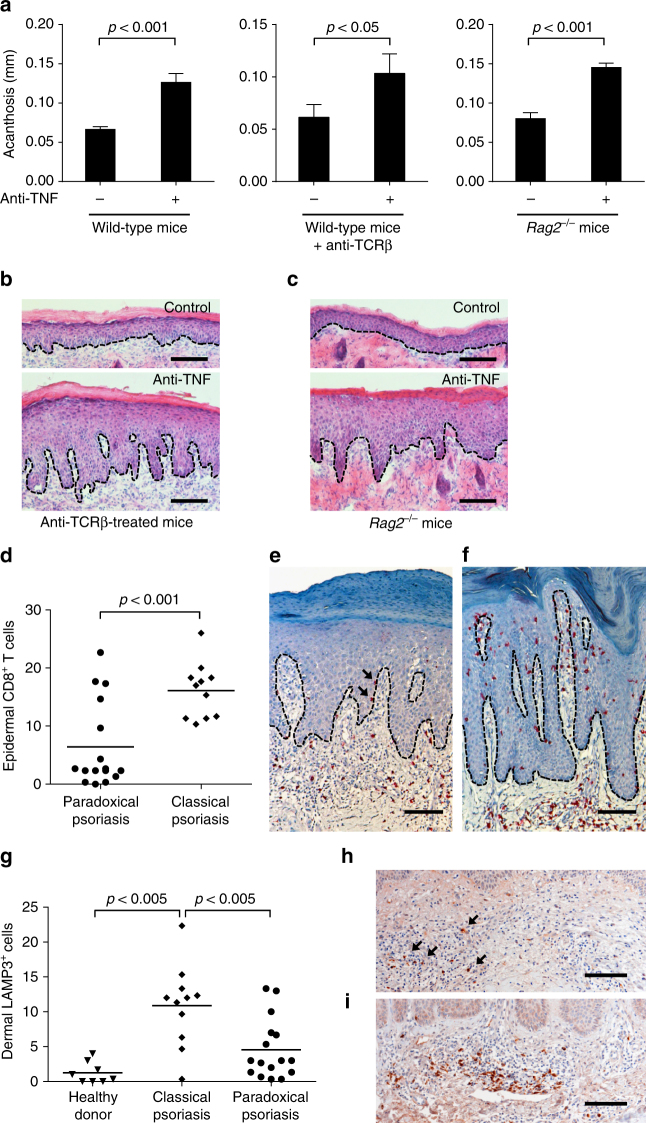
T-cell-independent induction of paradoxical psoriasis. **a** Quantification of acanthosis 7 days after mechanical injury of wild-type mice, wild-type mice treated with anti-TCRβ antibody, and *Rag2* ^−/−^ mice, all of which were treated with or without anti-TNF antibody. **b** Representative HE staining of skin 7 days after mechanical injury of mice treated with anti-TCRβ antibody alone or anti-TCRβ and anti-TNF antibodies combined. **c** Representative HE staining of skin 7 days after mechanical injury of *Rag2* ^−/−^ mice treated with or without anti-TNF antibody. **d** Number of epidermal CD8^+^ T cells per high-power field in skin lesions of patients with classical psoriasis and paradoxical psoriasis. **e**, **f** Representative CD8 staining of paradoxical psoriasis (**e**) and classical psoriasis (**f**). **g** Number of dermal LAMP3^+^ cells per high-power field in skin of healthy donors as well as in skin lesions of patients with classical psoriasis and paradoxical psoriasis. **h**, **i** Representative LAMP3-staining of paradoxical psoriasis (**h**) and classical psoriasis (**i**). Experiment depicted (in **a**–**c**) is representative for two independent experiments. Bar charts in **a** show mean values plus SEM of five mice each group. Dashed line in **b**, **c**, **e**, **f**, **h**, and **i** represents border between epidermis above and dermis below. Arrows point at intraepidermal CD8^+^ T cells in paradoxical psoriasis (**e**) or dermal LAMP3^+^ cells (**h**), respectively. All statistical analyses were performed with unpaired Student’s *t*-test. Anti-TCRβ = anti-T-cell receptor beta chain antibody

Type I IFN production by pDCs triggers classical psoriasis^35^ through activation of cDCs and expansion of autoimmune T cells. These pathogenic T cells are direct triggers of epidermal hyperproliferation and their persistence in the skin and circulation of psoriasis patients are responsible for chronicity and the recurrent disease course^19–21^. Because paradoxical psoriasis does not represent true psoriasis as it never relapses upon cessation of anti-TNF (Supplementary Table 1), we next asked whether T cells play a role in paradoxical psoriasis. We depleted conventional T cells in our paradoxical psoriasis mouse model using anti-TCR-beta antibody administration. T-cell-depleted mice treated with anti-TNF developed a psoriasis-like phenotype with increased acanthosis that was similar to non-depleted control mice treated with anti-TNF (Fig. 6a, b). Because unconventional T cells such as γ/δ-T cells have been implicated in the development of psoriasiform skin inflammation in mouse models^47^, we treated *Rag2* ^−/−^ mice, which are deficient of both conventional α/β-T cells and γ/δ-T cells, with anti-TNF. Similar to wild-type mice, anti-TNF-treated *Rag2* ^−/−^ mice developed a psoriatic phenotype with significantly increased epidermal thickness (Fig. 6a, c). These data indicate that neither conventional T cells nor γ/δ-T cells are required for the type I IFN-driven keratinocyte hyperproliferation. To investigate the role of T cells in human paradoxical psoriasis, we quantified CD8^+^ T cells infiltrating the epidermis, which represent the pathogenic T-cell subpopulation in psoriasis^28,36^. Compared to the large numbers of CD8 T cells present in the epidermis of classical psoriasis (*n* = 11), a significantly lower number of CD8^+^ T cells was present in the epidermis of paradoxical psoriasis (*n* = 16; Fig. 6d–f). CD8^+^ T cells were completely absent in normal skin of healthy donors (*n* = 5). Because mature cDCs in psoriatic skin represent the key stimulators of pathogenic CD8^+^ T cells to migrate into the epidermis, we next quantified mature cDCs in skin samples using the maturation marker LAMP3. We found a significantly increased number of LAMP3^+^ cDCs in the skin of classical psoriasis as compared to skin from healthy donors (Fig. 6g). In contrast, there were significantly fewer LAMP3^+^ cDCs in paradoxical psoriasis suggesting a lack of cDC maturation despite the increase type I IFN expression (Fig. 6g–i). Taken together, these data suggest that paradoxical psoriasis represents an overactive type I IFN-driven innate inflammation that does not lead to cDC maturation with consequent T-cell-mediated autoimmune response as in classical psoriasis.

## Discussion

This study identifies the pathophysiological mechanism underlying anti-TNF-induced paradoxical psoriasis. By comparing skin lesions of paradoxical psoriasis with classical psoriasis, we found a selective and uniform increase of type I IFN expression along with a marked dermal accumulation of pDCs. Using in vitro and in vivo models, we then demonstrated that anti-TNFs directly prolong the ability of pDCs to produce type I IFN. The resulting overexpression of type I IFNs is sufficient to drive the development of the psoriatic skin phenotype observed in paradoxical psoriasis, which, in contrast to classical psoriasis, is independent of T cells.

A link between anti-TNFs and increased type I IFN expression has been suggested by previous findings that anti-TNF therapy induces a type I IFN signature in blood of juvenile arthritis patients^48^ and can exacerbate lupus, a well-known type I IFN-driven autoimmune disease^49,50^. Using a combination of in vitro and in vivo studies, we now unravel the mechanism by which this occurs: TNF temporally controls and limits type I IFN expression by pDCs, and this effect can be reversed by anti-TNFs. Upon stimulation of pDCs, type I IFN production occurs first and is subsequently relayed by TNF production, which drives pDC maturation into DCs that lose the ability to produce type I IFNs^51^. Therefore, by promoting pDC maturation, TNF directly controls and limits the duration of type I IFN production by pDCs. On the other hand, blocking of TNF activity by anti-TNFs decreases pDC maturation and thereby prolongs the ability of pDC to produce type I IFN. Together our findings suggest a yin-yang model in which there is a temporal equilibrium between early type I IFN and late TNF expression^48^ that is shifted by TNF blockade toward a prolonged and excessive type I IFN response.

Our study shows that the type I IFN overproduction in paradoxical psoriasis is required for the development of a psoriatic skin phenotype. This finding raises questions about the differences between paradoxical psoriasis and classical psoriasis, which is also driven by an early type I IFN production by pDCs^35^. Our data show that unlike classical psoriasis, which is a T-cell-mediated autoimmune disease, development of paradoxical psoriasis is independent of T cells. Therefore, both paradoxical psoriasis and classical psoriasis are triggered by pDCs and type I IFN, but only classical psoriasis develops into a T-cell-mediated relapsing autoimmune disease. In contrast, paradoxical psoriasis fails to elicit an adaptive immune response and remains fixed in an ongoing pDC-driven innate immune response. These findings explain why there is no disease memory in paradoxical psoriasis while classical psoriasis is characterized by T-cell-mediated recurrent flare-ups. There are two possible explanations for the lack of T-cell autoimmunity in paradoxical psoriasis. In classical psoriasis, the type I IFN response is rapidly replaced by increasing levels of TNF, which is critical for the maturation of cDCs that stimulate T cells^46^. In the context of paradoxical psoriasis, TNF blockade inhibits the induction of mature cDC and subsequent T-cell activation, while magnifying type I IFN-driven innate inflammation. Another possibility is that paradoxical psoriasis patients lack genetic risk variants that drive and regulate T-cell autoimmunity. In fact, variants involving T-cell activation and Th17 differentiation, including *IL23A*, *IL23R*, *IL12B*, *HLA-Cw6*, *RUNX3*, *STAT3*, and *TRAF3IP2* genes have been identified in classical psoriasis^52,53^.

The mechanisms by which type I IFNs promote the psoriatic skin phenotype are currently unclear. Type I IFN itself does not induce keratinocyte proliferation nor is it responsible for the altered differentiation^54^. Most likely, type I IFN activates immune cells releasing cytokines that drive the development of a psoriatic phenotype. One possible link between type I IFN and keratinocyte hyperproliferation is IL22, which induces epidermal remodeling by promoting proliferation of keratinocytes^55,56^. Indeed, type I IFN drives *IL22* expression, as absence of type I IFN signaling completely abrogates induction of *IL22* expression in skin^43^. Accordingly, *IL22* is selectively upregulated in paradoxical psoriasis and significantly correlates with type I IFN expression. The cellular source of IL22 remains unclear. Because T cells do not play a role in paradoxical psoriasis, potential candidates include innate lymphoid cells (ILC3), natural killer cells^57^, mast cells^58^, and neutrophils^59^, which have all been reported to express IL22.

In addition to increased type I IFN expression, higher numbers of skin pDCs are observed in paradoxical psoriasis as compared to classical psoriasis. The increased pDC numbers is not a direct anti-TNF effect, but rather dependent on the type I IFN overexpression induced by TNF blockade. Although the exact mechanisms by which type I IFN drives pDC infiltration in paradoxical psoriasis remains to be elucidated, CXCR3 ligands induced by type I IFNs may prolong the recruitment of pDCs into the skin in a self-amplifying loop^44^.

Our study also identifies a new mouse model for the induction of a psoriasiform skin phenotype with acanthosis, basal keratinocyte hyperproliferation, and altered epidermal differentiation. This model displays the following three key features of human paradoxical psoriasis, which are clearly distinct from classical psoriasis. First, the psoriatic phenotype in this model is induced and not blocked by anti-TNFs; second, like in paradoxical psoriasis, the phenotype in this model is T-cell-independent, whereas classical psoriasis is a T-cell-mediated disease; and finally, the model shows sustained type I IFN expression, which is in line with the persistent type I IFN expression in the skin of paradoxical psoriasis but not classical psoriasis. An intriguing question is why anti-TNF is able to induce a psoriatic phenotype in wild-type mice and enhance type I IFN production by pDCs from blood of healthy donors, but only 2–5% of anti-TNF-treated patients develop paradoxical psoriasis. The future identification of specific genetic variants potentially involving pDC activation and/or type I IFN signaling may provide an explanation for an increased susceptibility of these individuals to develop paradoxical psoriasis in the context of anti-TNF treatment.

In conclusion, this study identifies the relevance of the temporal equilibrium of TNF and type I IFN (TNF-IFN yin-yang) in the pathogenesis of paradoxical psoriasis. While TNF controls type I IFN under steady-state conditions, anti-TNF treatment may tip the balance toward type I IFN ultimately driving the psoriatic phenotype in paradoxical psoriasis. In contrast to classical psoriasis, paradoxical psoriasis fails to turn into a T-cell-mediated autoimmune disease with a relapsing course but remains a drug-related side effect in which inflammation perpetuates in self-amplifying innate immune response. These findings provide the basis for the design of new strategies targeting pDCs and type I IFN for the treatment and prevention of paradoxical psoriasis.

## Methods

### Clinical data

This study was performed in accordance with the guidelines of the Declaration of Helsinki and was approved by the local ethics committee (Ethics Committee Vaud, swissethics). Clinical data of 25 patients with paradoxical psoriasis were collected at the Department of Dermatology, University Hospital CHUV, Lausanne (*n* = 16) and the Department of Dermatology, University Hospital of Zurich (*n* = 9) between 2011 and 2013. Paradoxical psoriasis was defined as newly appearing psoriasiform skin lesions under anti-TNF therapy despite response to treatment.

### Skin biopsies

Skin biopsies were taken from patients with paradoxical psoriasis or untreated classical plaque psoriasis after written informed consent was obtained. Human samples were fixed using 4% paraformaldehyde for immunohistochemistry (samples available from all 25 patients) or snap-frozen and stored at −80 °C for reverse transcription-PCR (RT-PCR) (cryomaterial available from 14 out of the 25 patients). Paraffin-embedded skin sections were deparaffinized, stained with anti-CD123 (BD Pharmingen), anti-LAMP3 (Sino Biological, 10527-RP02-50), or anti-CD8 (DAKO, C8/144B), and visualized using standard horseradish peroxidase technique. Frozen skin sections were fixed with 4% paraformaldehyde and stained for BDCA2 (anti-BDCA2-biotin, clone AC144, Miltenyi, plus streptavidin-Alexa488) and for CD123 (anti-CD123–phycoerythrin, clone 7G3, BD Biosciences). For quantitative RT-PCR, cDNA was synthesized using Superscript II reverse transcriptase (Invitrogen) and relative Gene expression was quantified using specific Taqman probes (Life Technologies, Supplementary Table 2) and expressed as 2^−ΔΔCT^ using *GAPDH* as endogenous control.

For immunofluorescence analyses of mouse tissue, cryopreserved skin samples were stained with anti-involucrin (Covance, PRB-140C-200, 1/400) or anti-Ki-67 (eBiosciences, SolA15, 1/1000) followed by labeled secondary antibody. For flow cytometry analysis, mouse skin was digested with Dispase (Sigma-Aldrich) and collagenase (Invitrogen), and stained with anti-B220 FITC (BD Pharmingen, RA3-6B2, 1/400), anti-CD45 PerCp-Cy5.5 (BD Pharmingen, 30-F11, 1/400), anti-CD11c PE (eBioscience, N418, 1/800), and anti-PDCA1 APC (Biolegend, 927, 1/400), anti-CD80 PE (BD Pharmingen, 16-10A1, 1/800), or anti-CD86 PE (BD Pharmingen, GL1, 1/800). For flow cytometry analyses of human pDCs, antibodies used include anti-CD123 APC (Biolegend, 6H6, 1/400), anti-BDCA2 PE (Miltenyi, AC144, 1/400), anti-BDCA4 APC (Miltenyi, REA-380, 1/400), anti-CD80 PE (BD Pharmingen, 16-10A1, 1/800), anti-CD83 FITC (eBioscience, HB15e, 1/400), and anti-HLA-DR (BD Pharmingen, G46-6, 1/400). Assessors were blinded for all histological quantifications.

### Cell culture

pDCs were purified from peripheral blood mononuclear cells obtained from blood buffy coats of healthy donors by Ficoll separation followed by enrichment using a CD304 Microbeads kit (Miltenyi Biotech). pDCs were cultured in RPMI 1640 + GlutaMAX (Gibco) supplemented with 10% fetal bovine serum and 1% penicillin/streptomycin and stimulated with 10 µg/ml human DNA (Biochain) complexed with 50 µg/ml LL37 (Proteogenix) with or without 1 µg/ml anti-TNF antibodies (Adalimumab, Humira), or 100ng/ml recombinant human TNF (RnD). After 48 h of culture, IFN-α was measured in cell-free supernatants by enzyme-linked immunosorbent assay (Mabtech).

### Mouse models

All animal experiments were performed according to institutional guidelines and Swiss federal and cantonal laws on animal protection. Ethical approval was obtained for all described experimentation according to regulations by the Federal Food Safety and Veterinary Office. Animals were maintained and bred in pathogen-free facilities. Age- (8–10 weeks old) and sex-matched mice were used for all experiments. Female wild-type Balb/c mice were purchased from Jackson Laboratory, *hBDCA2-DTR* (*hCLEC4C-DTR*) mice were bred at our facility. Skin injury was performed as previously described^43^. Briefly, mice were anesthetized and their lower backs shaved using clippers, and then depilated using the commercially available Veet cream. After cream removal with a paper tissue, 10 gentle strokes of commercially available tape (Scotch, 3M) were applied to the lower back. Dosage of antibodies applied was deduced from therapeutic use in humans and injected intraperitoneally as follows: 1500 µg of anti-TNF (infliximab, Remicade) at days −1 and 0; 200 µg of anti-TCRβ (BioXCell, h57-597) at days −2, 0, 2, and 4; and 250 µg of anti-IFNAR (BioXCell, MAR1-5A3) at days −1, 0, 1, and 3. We used Remicade because it was previously shown to efficiently block both human and mouse TNF^60^. However, as a positive control, we used a mouse-specific anti-TNF antibody (Supplementary Figure 8). As a negative control, we used an irrelevant human IgG antibody (Supplementary Figure 8). Effective blockade of type I IFN signaling by the anti-IFNAR antibody is demonstrated by the absence of type I IFN-response genes at day 7 after mechanical injury (Supplementary Figure 9). For pDC-depletion experiments, 120 ng of diphtheria toxin was injected intraperitoneally into *hBDCA2-DTR* mice at day −1. At indicated time points, injured skin was excised for histology, flow cytometry, and gene expression analysis.

### Statistics

Unpaired non-parametric Mann–Whitney *U*-test was used for analysis of human gene expression and histological analysis. To investigate an association between pDCs and type I IFN gene expression, the Spearman’s rank-correlation coefficient was calculated. For preclinical mouse data, Student’s *t*-test was used to perform statistical analyses. All testing was two-sided, and a *p*-value of <0.05 was considered to indicate statistical significance. All analyses were performed with GraphPad Prism 6.0.

### Data availability

All relevant data are available from the corresponding authors upon reasonable request.

## Electronic supplementary material

Supplementary Information
