# Supplementary material for: TNF blockade induces a dysregulated type I interferon response without autoimmunity in paradoxical psoriasis
Source: Nat Commun. 2018 Jan 2;9:25. doi: 10.1038/s41467-017-02466-4 (PMC5750213; doi:10.1038/s41467-017-02466-4)
Supplement: Supplementary file 1 — Supplementary Information [file 41467_2017_2466_MOESM1_ESM.pdf]

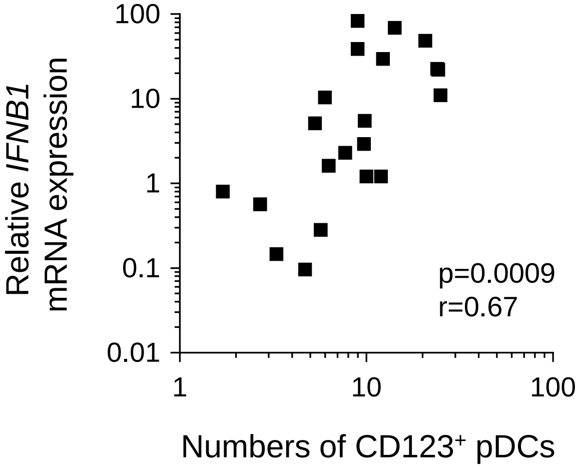

Supplementary Figure 1: Correlation of numbers of CD123-positive plasmacytoid dendritic cells with gene expression of *IFNB1* in skin lesions of paradoxical psoriasis. Dots represent individual patient. For statistical analysis, the Spearman's rank-correlation coefficient was calculated. pDCs = plasmacytoid dendritic cells.

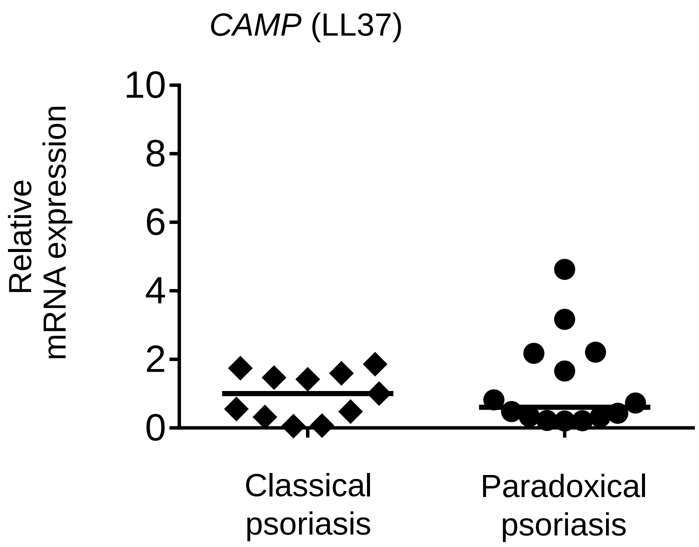

Supplementary Figure 2: mRNA expression analysis of *CAMP* (LL37) relative to GAPDH in skin lesions of paradoxical psoriasis compared to classical plaque psoriasis. Dots represent individual patient and horizontal bar denotes the median value. Data shown as mRNA expression level relative to mean expression in classical psoriasis (mean value for classical psoriasis was set at 1). Statistical analysis was performed with unpaired non parametric Mann-Whitney U test. CAMP = cathelicidin antimicrobial peptide.

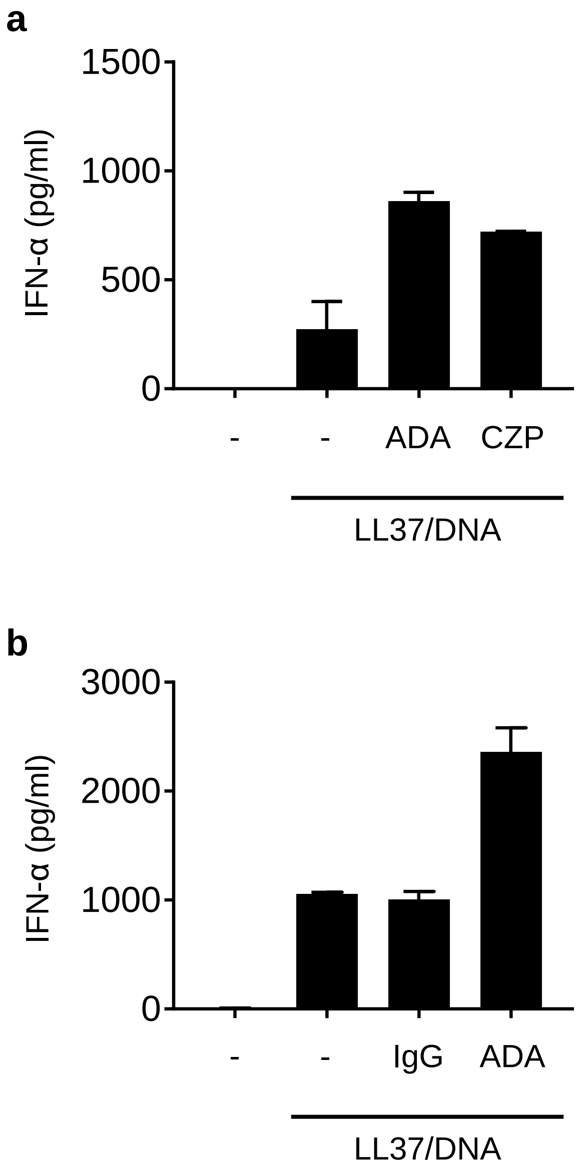

Supplementary Figure 3: IFN-α produced by plasmacytoid dendritic cells isolated from healthy volunteers, 48 hours upon stimulation with DNA-LL37 complexes with or without **(a)** anti-TNF agents – either a monoclonal antibody (adalimumab) or a FC-free antigen-binding fragment (Fab') of a monoclonal antibody (certolizumab pegol) or **(b)** an irrelevant human IgG antibody or a monoclonal anti-TNF antibody (adalimumab). Data depicts one representative experiment of five (a) or three (b) independent experiments with cells from different healthy volunteers for each experiment. ADA = adalimumab (Humira®), CZP = certolizumab pegol (Cimzia®). Error bars in represent S.D. of duplicate wells.

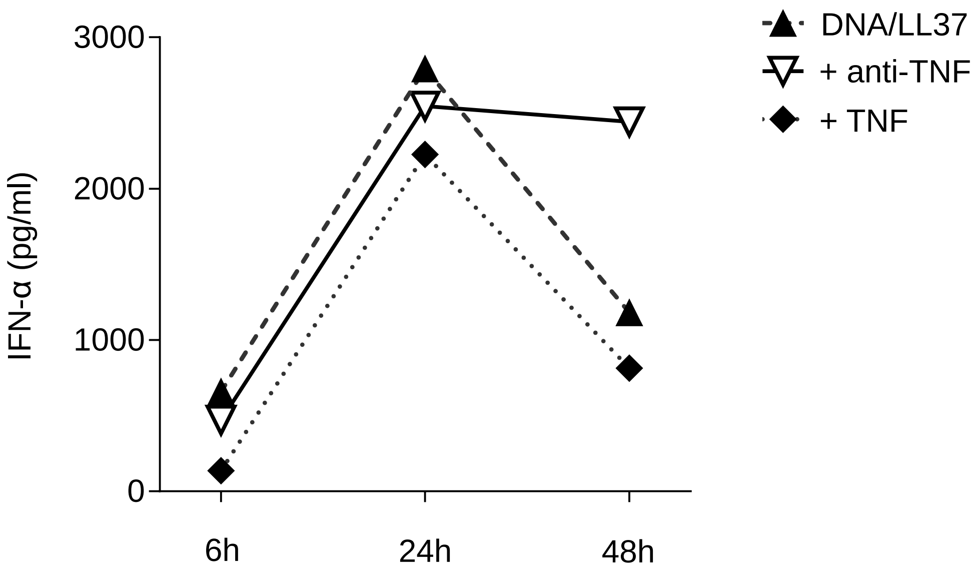

Supplementary Figure 4: IFN-α produced by plasmacytoid dendritic cells isolated from healthy volunteers and kept in culture for 6 hours, 24 hours or 48 hours respectively, upon stimulation with DNA-LL37 complexes either with anti-TNF antibodies or addition of TNF. Data depicts one representative of three independent experiments with cells from three different healthy individuals.

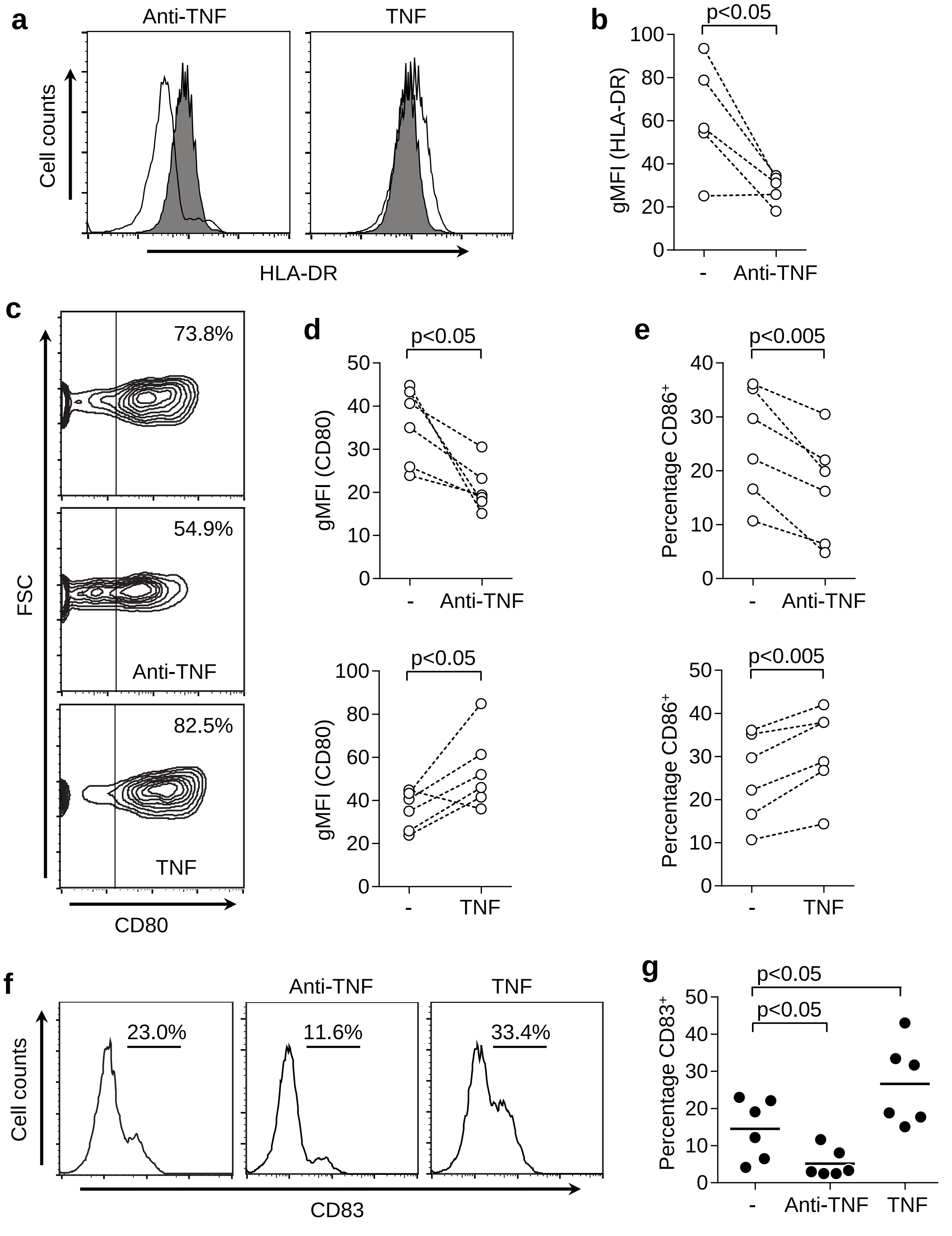

Supplementary Figure 5: HLA-DR (CD74) expression on plasmacytoid dendritic cells isolated from peripheral blood of healthy volunteers 48 hours upon activation with DNA-LL37 complexes with (black line) or without anti-TNF antibodies (gray shaded area), with (black line) or without TNF (**a**, **b**). Expression of co-stimulatory molecules CD80 (**c**, **d**) and CD86 (**e**) as well as maturation marker CD83 (**f**, **g**) on plasmacytoid dendritic cells isolated from healthy volunteers 48 hours after stimulation with 1μM CpGB in the presence or absence of anti-TNF or TNF. Experiments depicted in (**a**, **c**, and **f**) are representative for at least 5 independent experiments, each with blood from different healthy volunteers. Statistical analyses were performed with paired Student's t-test. gMFI = geometric mean fluorescence intensity, FSC = forward scatter.

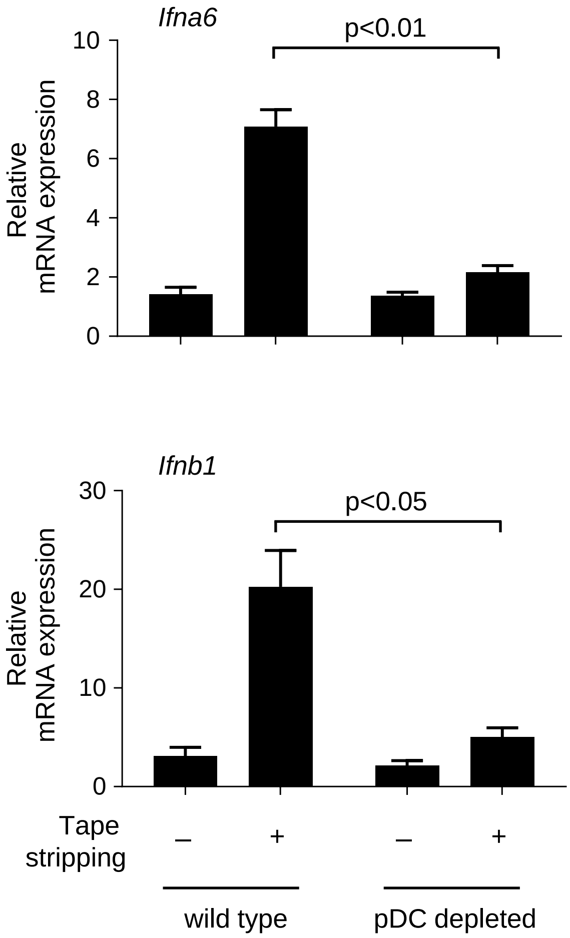

Supplementary Figure 6: Gene expression of the type I interferons *Ifna6* and *Ifnb1* in anti-TNF-treated mice in uninjured skin and upon mechanical injury in the presence or absence of plasmacytoid dendritic cells. The mean ± SEM is given and represent data from three mice. Experiment depicted is representative for at least 2 independent experiments. Statistical analyses were performed with unpaired Student's t-test. pDC = plasmacytoid dendritic cells.

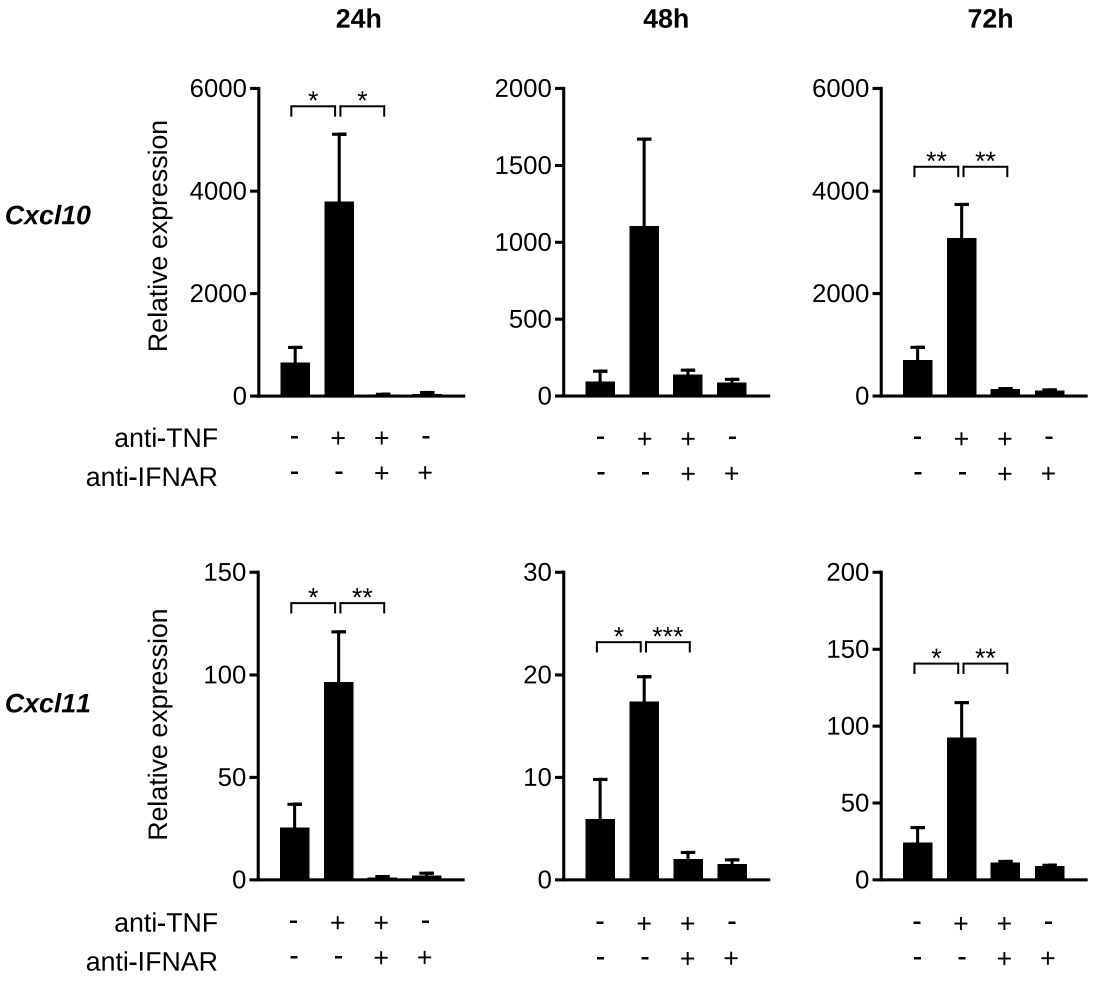

Supplementary Figure 7: Gene expression of chemokines *Cxcl10* and *Cxcl11* in the skin of mice treated with or without anti-TNF and/or anti-IFNAR antibodies 24 hours, 48 hours, and 72 hours after mechanical injury. The mean ± SEM is given and represent data from five mice. Experiment depicted is representative for at least 2 independent experiments. Statistical analyses were performed with unpaired Student's t-test. anti-IFNAR = anti-type I interferon receptor antibody. * $p<0.05$, ** $p\leq0.01$; ** $p\leq0.001$.

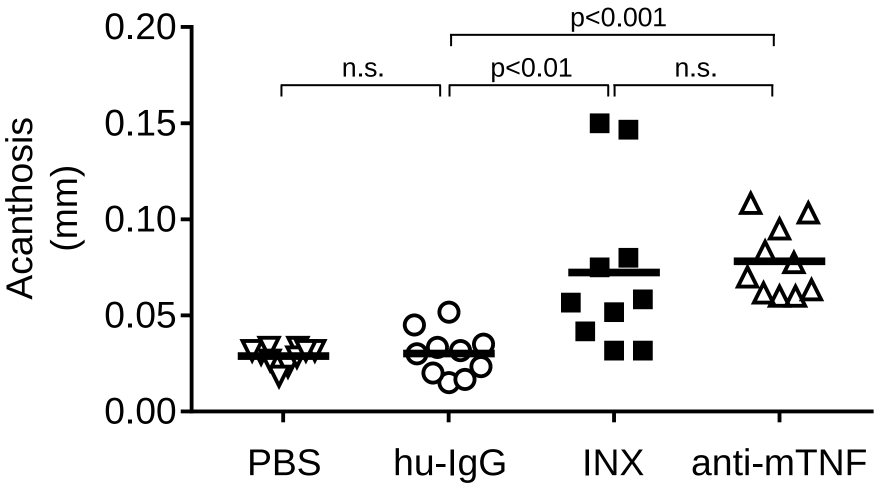

Supplementary Figure 8: Quantification of epidermal thickening (acanthosis) in mice treated with PBS, irrelevant human IgG antibody, the anti-TNF antibody infliximab (Remicade®), or an anti-mouse anti-TNF antibody 7 days after mechanical injury by tape stripping. Statistical analyses were performed with unpaired Student's t-test. PBS = phosphate buffered saline, hu-IgG = irrelevant human IgG antibody, INX = infliximab, anti-mTNF = anti-murine anti-TNF antibody, n.s. = not significant

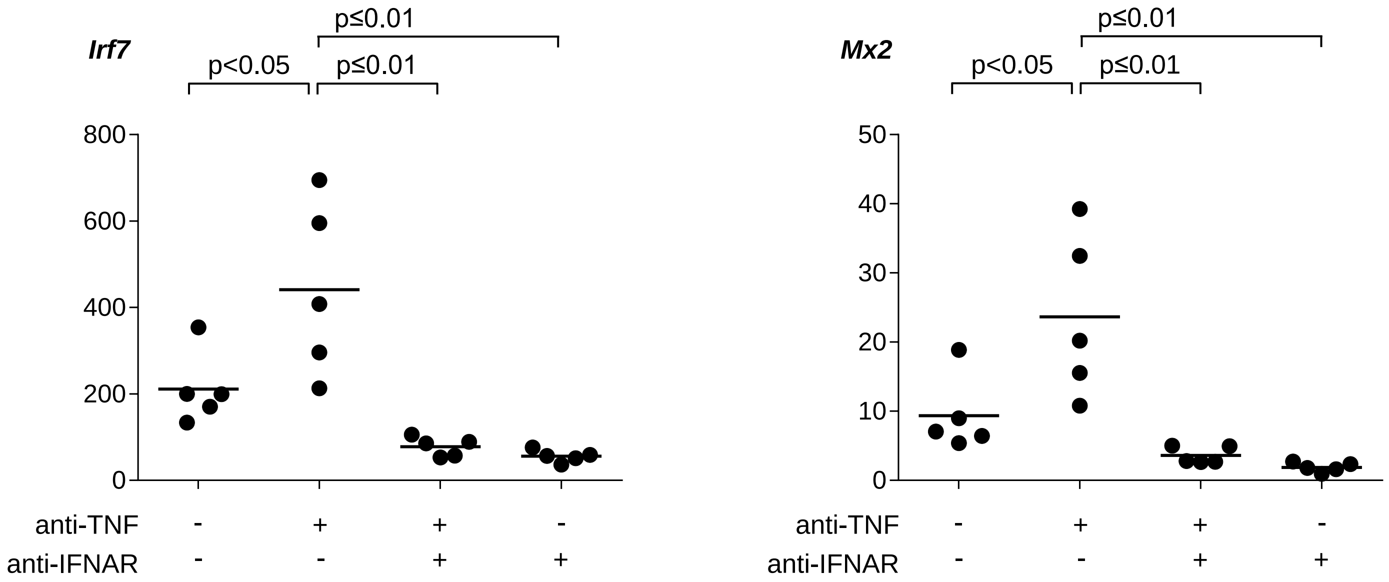

Supplementary Figure 9: Gene expression of the type I interferon response genes *Irf7* and *Mx2* in the skin of mice treated with or without anti-TNF and/or anti-IFNAR antibodies 7 days after mechanical injury. Statistical analyses were performed with unpaired Student's t-test. anti-IFNAR = anti-type I interferon receptor antibody.

Supplementary Table 1: Characteristics of the Patients with Paradoxical Psoriasis Induced by Anti-TNF Therapy.

Charactetristic	Age (years)	Sex	Diagnosis/Indication for anti-TNF	Anti-TNF agent	Duration of anti-TNF therapy until onset of paradoxical psoriasis	Localization**	Anti-TNF stopped or interrupted	Re-introduction of ant-TNF	Anti-TNF agent	Relapse	Therapy of paradoxical psoriasis***
Patient 1	22	M	Crohn's disease	Infliximab	18 months	B	Yes	Yes	Certolizumab	No	TS, TCI, SS
Patient 2	66	F	Plaque psoriasis and psoriatic arthritis	Adalimumab	2 months	P, B	Yes	Yes	Adalimumab	**Yes**	TS, SR, ustekinumab
Patient 3	36	M	Ankylosing spondylitis	Infliximab	5 months	P	No	Yes (anti-TNF continued)	Infliximab, Adalimumab	**Yes**	TS
Patient 4	48	F	Ankylosing spondylitis	Golimumab	2 months	P	Yes	Yes	Etanercept	No	TS, PUVA
Patient 5	68	F	Ankylosing spondylitis	Adalimumab	18 months	S, P, B	Yes	Yes	Etanercept	**Yes**	TS, PUVA, CsA
Patient 6	20	F	Crohn's disease	Infliximab	11 months	S, I	No	Yes (anti-TNF continued)	Infliximab	**Yes**	TS
Patient 7	60	M	Ankylosing spondylitis	Infliximab	8 months	B	No	Yes (anti-TNF continued)	Infliximab, Golimumab	No	TS
Patient 8	27	M	Crohn's disease	Infliximab	5 months	P	No	Yes (anti-TNF continued)	Infliximab, Adalimumab	No	TS, SR
Patient 9	70	M	Rheumatoid arhritis	Adalimumab	5 months	B	Yes	Yes	Etanercept	**Yes**	TS, SS
Patient 10	65	F	Plaque psoriasis and psoriatic arthritis	Etanercept	6 months	S, B	Yes	Yes	Etanercept	**Yes**	TS, SS, HXC, CsA
Patient 11	45	M	Ankylosing spondylitis	Adalimumab	3 years	S, I, B	Yes	Yes	Adalimumab	**Yes**	TS
Patient 12	39	F	Ankylosing spondylitis	Infliximab	2 months	S, P	Yes	No	None	No	TS
Patient 13*	64	F	Plaque psoriasis and psoriatic arthritis	Adalimumab	5 years	S, I, B	Yes	No	None	No	TS
Patient 14*	33	F	Crohn's disease	Adalimumab	24 months	I, B	Yes	No	None	No	TS
Patient 15	73	M	Plaque psoriasis	Etanercept	3 months	S, I, B	Yes	No (switch of class to ustekinumab)	None	No	PUVA
Patient 16	36	F	Palmoplantar psoriasis and psoriatic arthritis	Golimumab	3 months	S, P, B	Yes	No (switch of class to ustekinumab)	None	No	TS, PUVA, HXC
Patient 17	57	F	Plaque and palmoplantar psoriasis	Adalimumab	2 months	P, B	Yes	No	None	No	TS
Patient 18	42	F	Crohn's disease	Certolizumab	2 months	P, B	Yes	No	None	No	TS, TCI, SS, CsA
Patient 19	33	M	Plaque psoriasis and psoriatic arthritis	Infliximab	7 months	P, B	Yes	No	None	No	TS, TCI, MTX, CsA
Patient 20	55	F	Ankylosing spondylitis	Infliximab	2 months	S, P, I, B	Yes	No	None	No	TS, TCI, MTX
Patient 21	57	F	SAPHO/psoriatic arthritis	Adalimumab	3 weeks	S, P, B	Yes	No	None	No	TS, MTX
Patient 22	23	F	Crohn's disease	Infliximab	6 months	S, P, B	Yes	No	None	No	TS, SS, MTX
Patient 23	41	F	Ankylosing spondylitis	Infliximab	3 months	S, P, B	Yes	No	None	No	TS, SS, PUVA, UVB, CsA
Patient 24	15	F	Juvenile rheumatoid arhritis	Adalimumab	5 months	S, P, B	Yes	No	None	No	TS
Patient 25	25	F	Plaque psoriasis	Adalimumab	4 months	S, B	Yes	No	None	No	TS, CsA

* History of possible previous paradoxical psoriasis (infliximab and adalimumab respectively)

** S denotes scalp, P palmoplantar, I inverse, B rest of the body

*** TS denotes topical steroids, TCI topical calcineurin inhibitors, SS systemic steroids, SR systemic retinoids, PUVA psoralen + UVA therapy, CsA cyclosporine A, HXC hydroxychloroquine, MTX methotrexate

Supplementary Table 2: quantitative polymerase chain reaction (qPCR) probes.

Human probes	Mouse probes
TNF: Hs00174128_m1	*Ifna6*: Mm01703458_s1
IL6: Hs00174131_m1	*Ifnb1*: Mm00439552_s1
IFNA2: Hs00265051_s1	*Cxcl10*: Mm00445235_m1
IFNB1: Hs01077958_s1	*Cxcl11*: Mm00444662_m1
IL36G: Hs00219742_m1	*Irf7*: Mm00516793_g1
IL12A: Hs01073447_m1	*Mx2*: Mm00488995_m1
IL23A: Hs00372324_m1	
IL8: Hs00174103_m1	
IL1B: Hs01555410_m1	
IL17A: Hs00174383_m1	
IL17F: Hs01028648_m1	
IL17C: Hs00171163_m1	
IL22: Hs01574154_m1	
IL26: Hs00218189_m1	
IFNG: Hs00989291_m1	
IL4: Hs00174122_m1	
IL10: Hs00961622_m1	
